# A network analysis study on the relationship between generalized anxiety symptoms, big five personality and perceived social support of Chinese residents during COVID-19

**DOI:** 10.3389/fpubh.2025.1548718

**Published:** 2025-02-13

**Authors:** Jiaqin Yang, Xiaotong Man, Chunlei Liu

**Affiliations:** ^1^School of Psychology, Qufu Normal University, Qufu, China; ^2^Key Laboratory of Modern Teaching Technology (Ministry of Education), Shaanxi Normal University, Xi’an, China

**Keywords:** generalized anxiety disorder, agreeableness, perceived social support, network analysis, network comparison

## Abstract

**Introduction:**

Under the background of COVID-19, people’s mental health problems are concerned by researchers. Network analysis is a new method of exploring the interactions between mental health issues at the symptom level. This study investigates the network structure of generalized anxiety symptoms among Chinese residents during the COVID-19 pandemic from the perspective of “society-family-personality,” and explores its relationship with the Big Five personality traits and perceived social support.

**Methods:**

A multi-stage random sampling cross-sectional survey was conducted in 120 cities across China Mainland from July 10, 2021 to September 15, 2021, based on the PBICR database. The Big Five Scale (BFI-10), Perceived Social Support Scale (PSSS), and Generalized Anxiety Scale (GAD-7) were used for measurement. Pearson correlation analysis was used to examine the variables mentioned in this research, and network analysis was used to estimate the psychopathological network of the three variables.

**Results:**

A total of 11,031 subjects were included in the study, with 17% of individuals suffering from severe generalized anxiety symptoms. The results showed a correlation between the three research variables, and it was found that perceived social support in both dimensions and agreeableness of the Big Five personality traits were at the center of the network, with a significant impact on the overall network. There is a positive correlation between agreeableness and family support, but a negative correlation with generalized anxiety symptoms. Agreeableness serves as an indicator linking the other two variables; No significant gender differences were found through gender network testing.

**Conclusion:**

According to this study, we believe that interventions in family atmosphere and social interaction can be used to prevent symptoms of generalized anxiety disorder. The limitation of this study is that it cannot determine the causal relationship between variables and its generalizability in general contexts has not been confirmed. Future research can further explore its directionality based on this study and consider the influence of cultural factors to extend its applicability to other backgrounds.

## 1 Introduction

Generalized anxiety disorder (GAD) is a specific type of mental disorder, which is characterized by distractibility and uncontrollable nervousness with no apparent trigger (1). GAD has a high lifetime prevalence, which is often associated with low levels of self-perceived health, low quality of life scores, and impaired social functioning. It has brought significant negative consequences for public health (2).

Perceived social support refers to an individual’s subjective perception of the help and support provided to them by their social network (3). According to different sources, perceived social support can be categorized into two types: perceived intra-family support and perceived extra-family support. It has been found that perceived social support plays an important role as a stress buffer when people are under high levels of stress (4–6). That is, it is thought to provide a protective role as individuals cope with stressful experiences by fostering a more positive interpretation of events, which, in turn, can help to minimize an individual’s anxiety and stressful experiences (4).

After decades of empirical testing, the Big Five model has become a well-recognized personality trait model in psychology, encompassing the five traits of openness, conscientiousness, extraversion, agreeableness, and neuroticism. The current study investigated whether any of the Big Five personality traits interacted to predict perceived support. Extraversion, neuroticism, and openness predicted overall social support, and these three traits interacted to predict perceived social support. That is, at low levels of extraversion, low neuroticism was associated with greater perceived social support, independent of openness. However, as extraversion increased, the combination of low neuroticism and low openness was associated with the greatest degree of perceived social support. High extraversion, high neuroticism, and low openness were associated with the lowest levels of perceived support (7). A study has also been conducted to examine the effects of Big Five personality traits, health anxiety, and COVID-19 psychological distress on generalized anxiety and depressive symptoms in the context of the COVID-19 epidemic. This study noted that extraversion, agreeableness, conscientiousness, and openness were negatively correlated with generalized anxiety and depressive symptoms, and neuroticism, health anxiety, and two indicators of the COVID-19 distress were positively correlated with generalized anxiety and depressive symptoms (8).

There are fewer current studies on the relationship between perceived social support, GAD, and personality traits, and the present study aimed to explore the network structure of all three, to broaden the scope of existing research on generalized anxiety, to study the psychopathological network of generalized anxiety symptoms in a social-family-personality perspective, to identify its core symptoms, and to provide recommendations for its preventive intervention.

## 2 Methods

### 2.1 Data sources

In this study, we use the database of “Psychology and Behavior Investigation of Chinese Residents, PBICR” of 2021, which was conducted from July 10, 2021 to September 15, 2021 (9). Based on the results of the Seventh National Population Census in 2021, quota sampling (quota attributes are gender, age, and urban/rural distribution) was conducted on 120 urban residents, so that the gender, age, and urban/rural distribution of the samples basically conformed to the demographic characteristics of the population. Excluding Hong Kong, Macao and Taiwan, there were 11,031 participants, including 5,033 males and 5,998 females. The subjects covered all age groups, of which 9.6% were 18 years old and below, 18.3% were 19–25 years old, 22.7% were 26–35 years old, 18.7% were 36–45 years old, 17.4% were 46–55 years old, 5% were 56–65 years old, and 8.3% were over 65 years old. 8.3% of the total number of subjects were over 65 years old. The investigators contacted potential participants from 23 provinces, 5 autonomous regions and 4 municipalities directly under the central government either face-to-face or by phone. To ensure the quality of the survey, participants were recruited openly from each municipality and trained in standardized procedures and unforeseen circumstances for the technique.

### 2.2 Questionnaires

#### 2.2.1 Big five personality inventory

We use the Chinese 10-item Big Five Personality Inventory (BFI-10) (10) to assess personality traits including extroversion, agreeableness, conscientiousness, neuroticism, and openness. The BFI-10 consists of 10 entries in a Likert format of 1 (strongly disagree) to 5 (strongly agree). Each personality trait score is derived from 2 items, with all scores ranging from 0 to 10. Higher total scores for each trait indicate a higher degree of personality trait of the individual.

Previous studies have shown considerable reliability and good validity for all aspects (11). In the current test, the Cronbach ‘s alpha of the questionnaire was 0.66. Hair et al. (12) pointed out that when the measured indicators of variables are less than 6, Cronbach’s *α* coefficient is greater than 0.6, indicating that the scale is reliable.

#### 2.2.2 Perceived social support scale

The Perceived Social Support Scale (PSSS) (13) was used, which consists of 12 items to measure the degree of social support perceived by an individual from family, friends, and significant others, and was scored on a five-point scale in the current study (1 = Strongly Disagree, 5 = Strongly Agree). The Cronbach ‘s alpha of the questionnaire was 0.96 on the current test.

#### 2.2.3 Generalized anxiety scale

The GAD-7 consists of seven questions related to symptoms of generalized anxiety disorder over the past 2 weeks, categorized into four levels: ‘never’, ‘a few days’, ‘more than half the time’ or “almost every day.” Items include: nervousness, inability to stop worrying, excessive worrying, restlessness, difficulty relaxing, easy irritation, and fear of something terrible happening (14). The total score on the GAD-7 ranges from 0 to 21, with higher scores indicating stronger associations with anxiety-induced functional impairment. The Cronbach’s alpha for the questionnaire was 0.96 on the current test.

### 2.3 Data analysis

The data analysis method includes two parts: descriptive statistical analysis and network analysis. Before analysis, we standardized the data. First, descriptive statistics were used to analyze all the data in this study to explore the role of participants’ basic information and demographic variables. Second, network analysis was conducted using R 4.4.0 to explore the network structure of the dimensions/items of generalized anxiety symptoms, Big Five personality traits, and perceived social support. The steps of the network analysis followed the standardized guidelines published by Epskamp et al. (15), and the analysis consisted of five parts: network estimation, visualization of the network, estimation of the centrality index, network comparison, and estimation of network accuracy and stability. The two levels of the network are complementary. Item level network analysis can be used to test the relationship between the items of the self-report scale, and gain insight into the relevance and importance of each item (symptom) in the network, which can help us understand the research problem more comprehensively.

#### 2.3.1 Network estimation

According to the standardization guide of network analysis steps published by Epskamp et al. (15), the bias correlation network estimation of the sample uses the qgraph package in the R language software, in which the circular nodes indicate the dimensions/questions, and the connecting lines between the nodes are called edges, whose thickness indicates the size of the bias correlation coefficients. In this study, the following procedure was performed for each biased correlation network: in the first step, a Gaussian graphical model estimation (16) was performed, which estimates the pairwise correlation parameter between all the nodes; in the second step, the least absolute contraction and selection operator (17) was used to avoid false positive associations. This procedure is a regularization technique that sets edges with smaller associations in order to identify edges associated with them more carefully, and thus identify potential network structures more precisely (18).

#### 2.3.2 Network visualization and centrality measures

Separate visualization estimates of the networks were performed. All networks were visualized using the Fruchterman-Reingold algorithm (19). In this study, the positively correlated edges were set to blue, and the negatively correlated edges were set to red, and the thicker lines of the edges indicated the stronger connection between two nodes. In the network, nodes that are clustered together indicate stronger connections between them or more connections between them. Centrality indices are the core for quantitatively evaluating the importance of a node, and are used to elucidate the degree of centrality of a node in the entire network. Centrality measurements for networks include strength, betweenness, closeness and expected influence. Strength centrality refers to the sum of the weighted values of all connections associated with a node, the higher the strength centrality index, the higher its position and importance in the center of the network. Betweenness refers to the degree to which a node is located in the shortest path of the two nodes in the network that are connected to each other. If a node acts as an “intermediary” the more times, the stronger the intermediary of this node. Without this node, many two connected nodes will not be able to connect. Closeness refers to a node to all other nodes of the sum of the distance. This indice is the reciprocal of the path length, which means that the shorter the path length, the stronger the closeness. This indice emphasizes the proximity value of a node in the network; the greater the closeness, the closer the node is to all other nodes and the more centrally located it is. Expected influence refers to “the sum of the weighted values (both positive and negative) of the direct connections to other nodes, and is a more accurate predictor of a node’s influence on the network when a network contains both positive and negative edges.

#### 2.3.3 Network comparison

To explore the gender differences of networks, this study compares the networks of different genders from the perspectives of global invariance and local invariance by the method of permutation test. Network comparisons were analyzed using the NetworkComparisonTest package in R software for global invariance test and local invariance test, and the significance level was set at 0.05, and results less than 0.05 were considered to be significant (20). The global invariance test is divided into two parts: the network structure invariance test and the overall network strength invariance test. The network structure invariance test explores the maximum difference between the absolute values of the edge weights in each network, while the network overall strength invariance test explores the difference between the sum of the absolute values of all the edge weights in each network. The local invariance test examines the difference between the edge weights and the centrality indices of each node in each sample network and is corrected using the Holm-Bonferroni algorithm (21).

#### 2.3.4 Network accuracy and stability estimation

In this study, the accuracy and stability of the network were estimated by bootnet package in R language software (15). The accuracy of the edge weights in this study was estimated by the 95% confidence interval of the bootstrap edge weights, and the smaller the area covered by the confidence interval, the more accurate the edge estimation. When a certain proportion of subjects are deleted and node centrality is re-estimated through the subset construction procedure, and the correlation between this centrality and the original centrality index reaches 0.7, the proportion of deleted subjects is defined as the Centrality Stability Coefficient (CS). When this coefficient is greater than 0.25, it means that the stability is within the acceptable range, and when the coefficient is greater than 0.5, it means that the stability is good.

## 3 Results

### 3.1 Network analysis of the items

#### 3.1.1 Correlation analysis of each item

**Figure 1:**
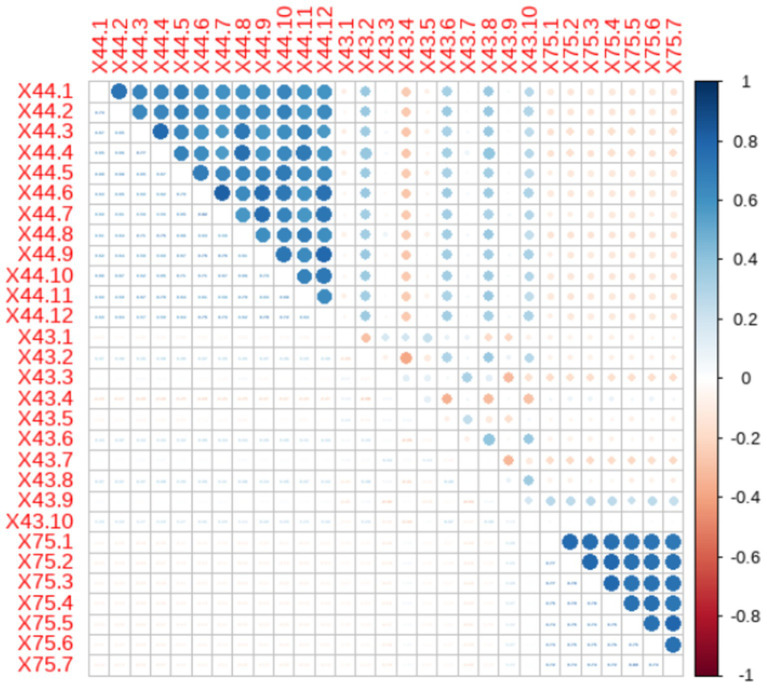
Correlation matrix for each question item.

The correlation analysis of each item was conducted, and the results are shown in Figure 1. There is a certain correlation between the items within each scale. And there is a strong positive correlation among the items in the Perceived Social Support Scale, both positive and negative correlations among the items in the Big Five Personality Scale, and a strong positive correlation among the items in the Generalized Anxiety Scale. There was some degree of correlation between some of the items in the different scales. In this study, |R| ≥ 0.6 is defined as strong correlation, and 0.4 ≤ |R|<0.6 is defined as medium correlation|R|<0.4 is a weak correlation. 18.97% of the items have strong correlation, 78.57% of the items have moderate correlation, and the rest have weak correlation.

#### 3.1.2 Network estimation

**Figure 2:**
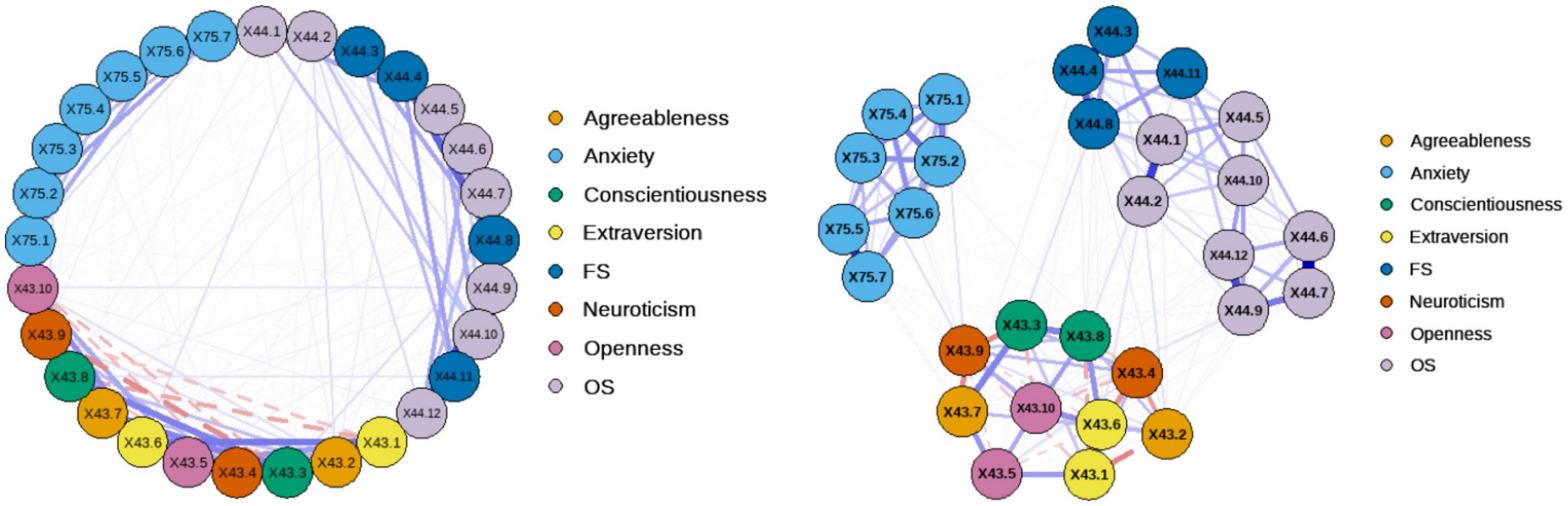
Network analysis of the question items (ring diagram on the left, circle diagram on the right).

To explore the network structure and core symptoms of the psychological pathological network under investigation, network estimation was first performed on the items of the aforementioned three variables. A regularized network with 29 nodes and 242 edges was estimated for the internal structure of the item network, in which 242 edges had non-zero weights (mean weight 0.09). In the item network, it can be seen that the variables are internally clustered together with large correlations, and that of the three scale items, both Perceived Social Support and Generalized Anxiety Symptoms are related to the Big Five personality (see Figure 2). As shown in Figure 2: there are both positive and negative correlations between each node and the others. Among them, X75.1, X75.2, and X75.4 in generalized anxiety are strongly connected. There is a close connection between X43.3, X43.7, and X43.8 in Big Five personality. There is a close connection between X44.1 and X44.2, X44.12 and X44.9, and X44.6 and X44.7 belonging to perceived social support. Different items belonging to the same dimension are more likely to cluster together, but items between dimensions are also more closely connected to each other.

#### 3.1.3 Centrality indicators

**Figure 3:**
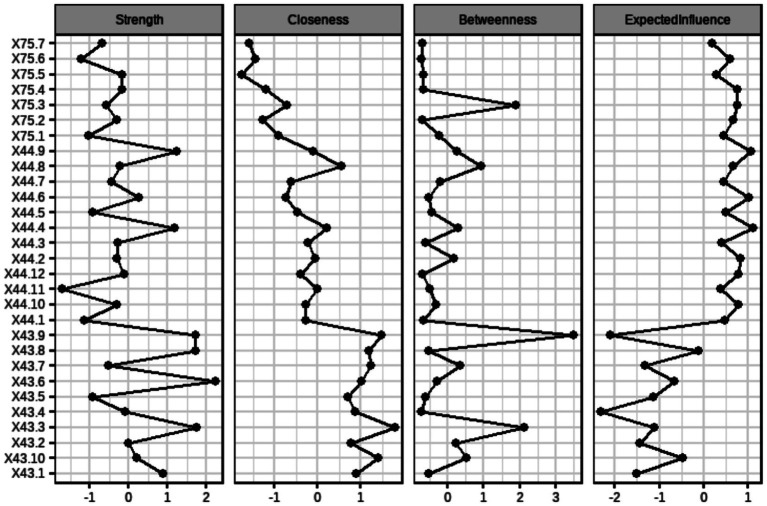
Centrality indicators after standardization of each question item.

The standardized network centrality indicators for the aforementioned network are illustrated in Figure 3. The strength centrality indicator of X43.6, which measures extraversion, is the highest in this item network, indicating that it is at the center of the network and is of high importance to the network as a whole, followed by X43.9, which measures neuroticism, and X43.8、X43.3, which measures conscientiousness. In addition, X43.3 shows the highest closeness indicators, followed by X43.9. As for betweenness centrality, the top two nodes are consistent with closeness, indicating that these two nodes are closer to all other nodes and are located in the center. In terms of expected influence, X43.9 shows the highest, indicating that this node has the greatest influence on the question-item network.

In the stability test of network centrality, the stability coefficient of the strength centrality of the item network is 0.67, the stability coefficient of the tightness is 0.75, and the stability coefficient of the mediator centrality is 0.52. The CS coefficients are in the acceptable range, the centrality indexes of each item are more stable, and the overall fit is better, as shown in the Supplementary Figures 1–5.

### 3.2 Network analysis of various dimensions

#### 3.2.1 Correlation analysis

**Figure 4:**
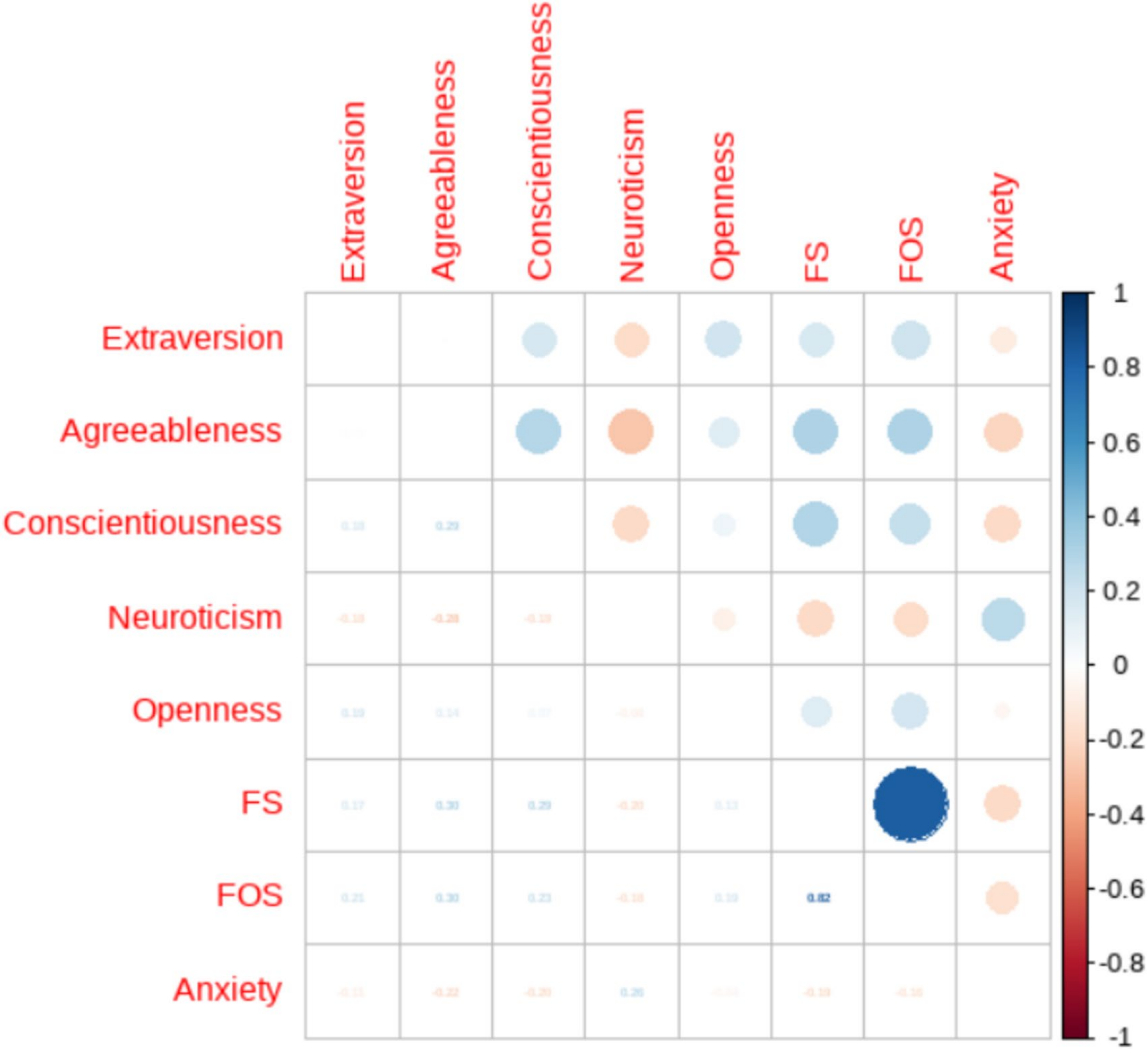
Correlation matrix diagram for each dimension.

Correlation analysis was conducted on each dimension, and the results are shown in Figure 4. There is a certain correlation between the dimensions of each variable. 3.57% of the dimensions have strong correlation, and the rest are weak correlation. In the dimension of perceived social support, there is a strong negative correlation between support within and outside the family, while both are strongly positively correlated with the total score of perceived social support. Generalized anxiety disorder is positively correlated with neuroticism in the Big Five personality traits, and negatively correlated with agreeableness, conscientiousness, and extroversion. There are both positive and negative correlations between the five personality traits in the Big Five personality traits.

#### 3.2.2 Network estimation

**Figure 5:**
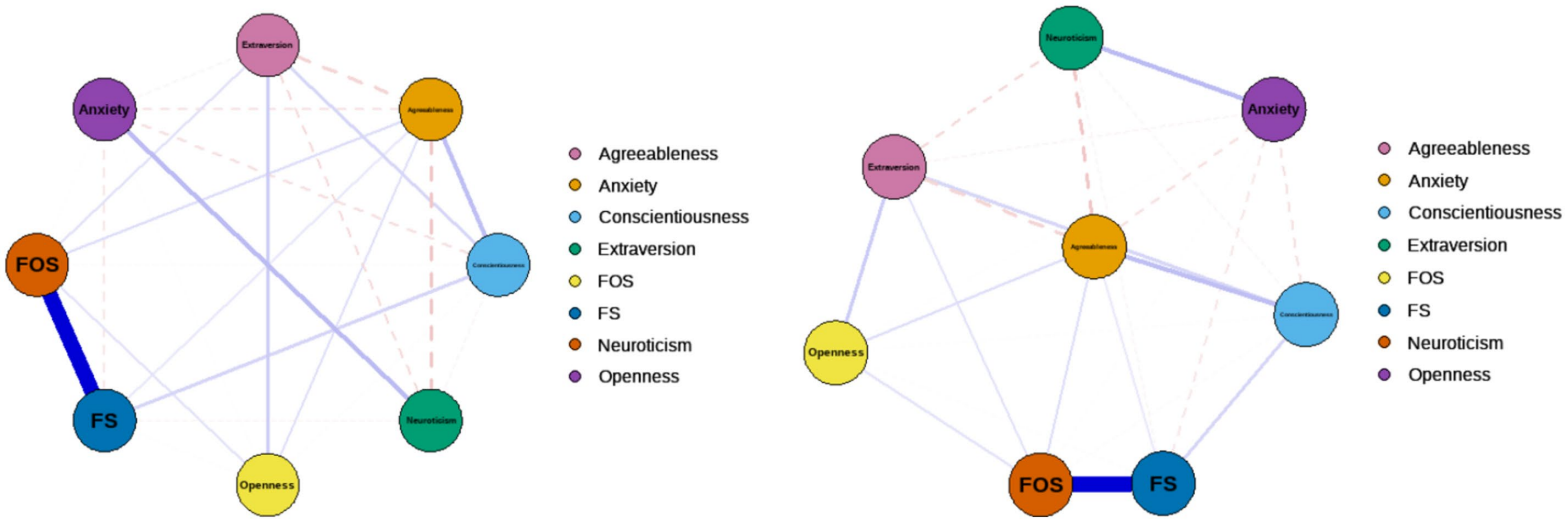
Network analysis by dimension.

To explore the network structure and core dimensions of the Big Five personality, Perceived Social Support, and Generalized Anxiety Symptoms, network estimation of the dimension of the above three variables was conducted. A regularized network containing 8 nodes and 25 edges was estimated for the internal structure of the dimensional network, where the 25 edges had non-zero weights (mean weight of 0.10). In the dimensional network, a link between the Big Five personality dimensions and generalized anxiety symptoms can be seen, and a positive correlation between perceived social support in the form of intra-family support, extra-family support, and agreeableness can be seen (see Figure 5). Intra-family support and extra-family support interact very strongly with each other. While generalized anxiety is negatively correlated with conscientiousness, agreeableness, and intra-family support, and is positively correlated with neuroticism. Conscientiousness was positively correlated with intra-family support and agreeableness. It can be hypothesized that generalized anxiety symptoms and agreeableness are at the core of the dimensional network.

#### 3.2.3 Indicators of centrality

**Figure 6:**
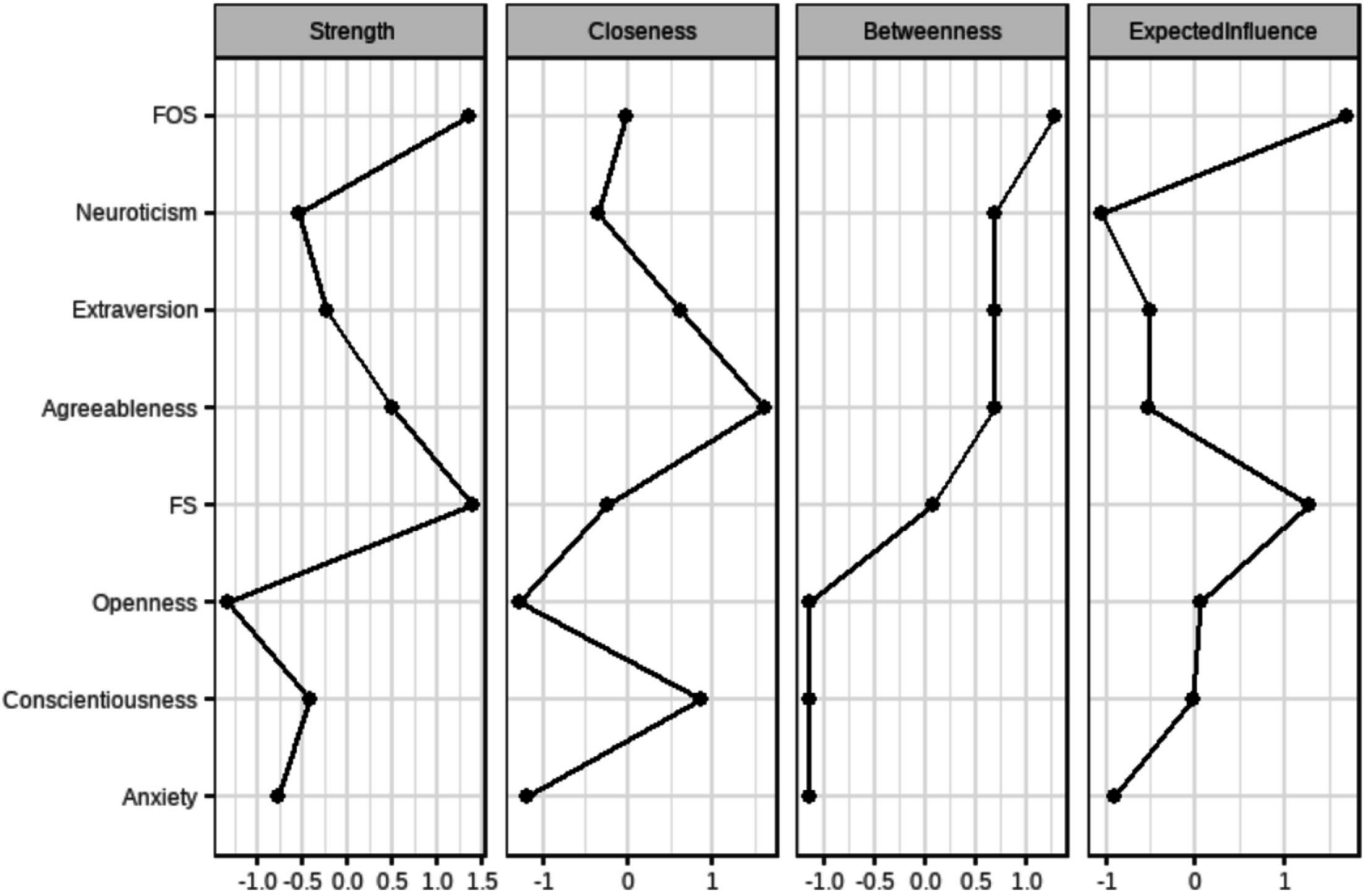
Centrality indicators after normalization for each dimension.

The standardized centrality indicators of the Perceived Social Support, Big Five Personality, and Generalized Anxiety Symptoms dimensional networks are shown in Figure 6, where the centrality indicators were analyzed separately in the dimensional networks. Perceived social support has the highest centrality in terms of the strength of family support, indicating that it is at the center of the network and is of high importance to the network as a whole, followed by the extra-family support dimension. In terms of closeness, the closeness of agreeableness was the highest, followed by conscientiousness. The betweenness centrality of perceived extra-family support is the highest, indicating that the node is closer to all other nodes and is located in the center. Perceived extra-family support has the highest expected influence. This suggests that the dimensions of perceived social support in this network and agreeableness, and conscientiousness are at the center of the network and have a greater influence on the overall network.

On the network centrality stability test, the stability coefficients of strength centrality and closeness of this dimensional network are 0.75, and the centrality stability coefficient of betweenness is 0.59. The CS coefficients are all greater than 0.5, which indicates that the network is stable, as shown in Supplementary Figures 6–10.

#### 3.2.4 Bridge connection indicators

**Figure 7:**
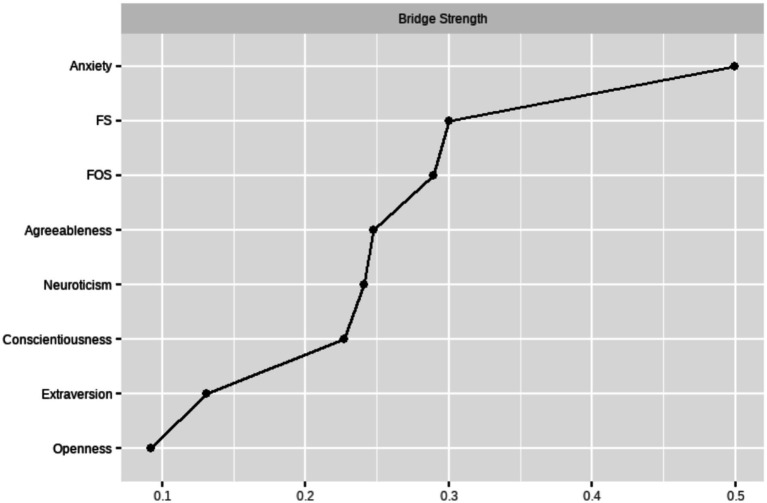
Bridge connection metrics by dimension.

Bridge strength refers to the sum of weighted absolute values directly connected to other disease nodes, which can reflect symptoms that increase the risk of transmission to other diseases, known as “bridge symptoms.” To test the bridge connection index of each dimension, a bridge connection graph was created, and it was found that the bridge connection index of generalized anxiety was the strongest (Figure 7). Visualization of the distribution of bridge connection strengths is shown in Supplementary Figure 11.

#### 3.2.5 Gender network comparison

**Figure 8:**
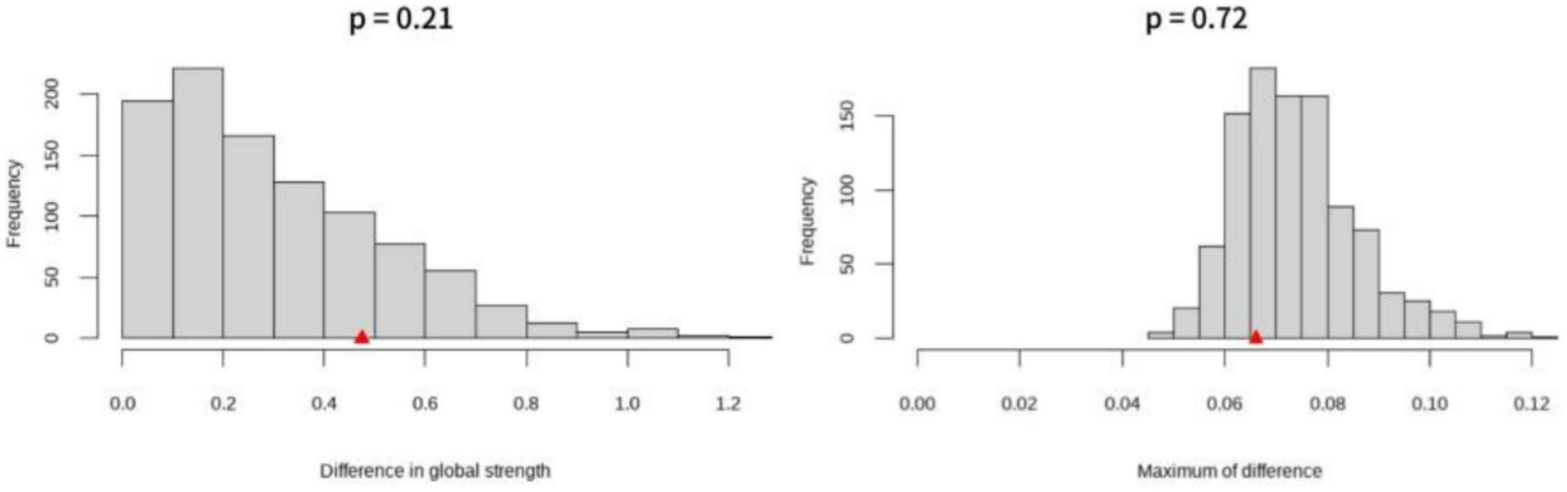
Differences in global strength and network structure of gender networks.

The network models for females (*n* = 5,998) and males (*n* = 5,033) were found to have no significant difference in global intensity (*p* = 0.21), and no significant difference in minimum value difference (*p* = 0.72). That is, there was no significant difference in the gender network (Figure 8).

## 4 Discussion

The network analysis of perceived social support, personality factors and generalized anxiety in this study provides evidence on the influencing factors of the development of generalized anxiety in the context of the COVID-19 epidemic. Through the analysis of item network and dimension network, we can see the core dimensions and indicators in the network directly. We find that agreeableness and perceived social support play an important role in it, and expand the research on generalized anxiety.

In this study, it was found that agreeableness and perceived social support are at the center of the psychological pathological network of generalized anxiety, perceived social support, and Big Five personality. And there is a positive correlation between the two dimensions of perceived social support and agreeableness, and there is a negative correlation between generalized anxiety symptoms and agreeableness, which serves as an indicator linking the other two variables. There is a strong positive correlation between generalized anxiety symptoms and neuroticism. And there was no significant difference between male and female. In this study, the percentage of individuals with severe generalized anxiety was 17%, which was higher than the prevalence during the period without the COVID-19 epidemic.

The limitation is that this study uses a cross-sectional design, which limits causal inference. In the future, longitudinal tracking tests can be used to explore the causal relationship. The gender difference in this study is not significant, which may be affected by specific cultural factors in the research background. The study was conducted in China. Chinese culture pays more attention to collectivism, especially during the period of the COVID-19 epidemic (22). Future research can further consider the influence of cultural factors and extend its applicability to other situations. Finally, in terms of sample selection, the population can also be classified in the future to explore whether there will be differences in generalized anxiety symptoms and its influencing factors under different age, occupation and other conditions.

## 5 Conclusion

Based on a large sample of Chinese residents, this study uses network analysis to estimate and analyze the network of generalized anxiety and its influencing factors, and provides a new analytical perspective for the study from the perspective of “society-family-personality.” Through network analysis, it can be found that agreeableness and perceived social support are at the center. There is a potential tendency for different personality tendencies to have an effect on generalized anxiety, and there may be an effect of perceived social support on agreeableness. The role of perceived social support as a central node is an important discovery, but its interaction mechanism with generalized anxiety and personality traits needs to be further explored. In terms of intervention and prevention of generalized anxiety, family atmosphere and social interaction can be tried.

## Data Availability

The original contributions presented in the study are included in the article/Supplementary material, further inquiries can be directed to the corresponding author.
