# Supplementary material for: A network analysis study on the relationship between generalized anxiety symptoms, big five personality and perceived social support of Chinese residents during COVID-19
Source: Front Public Health. 2025 Feb 13;13:1548718. doi: 10.3389/fpubh.2025.1548718 (PMC11864928; doi:10.3389/fpubh.2025.1548718)
Supplement: Supplementary file 1 [file Supplementary_file_1.docx]

Supplementary Figures


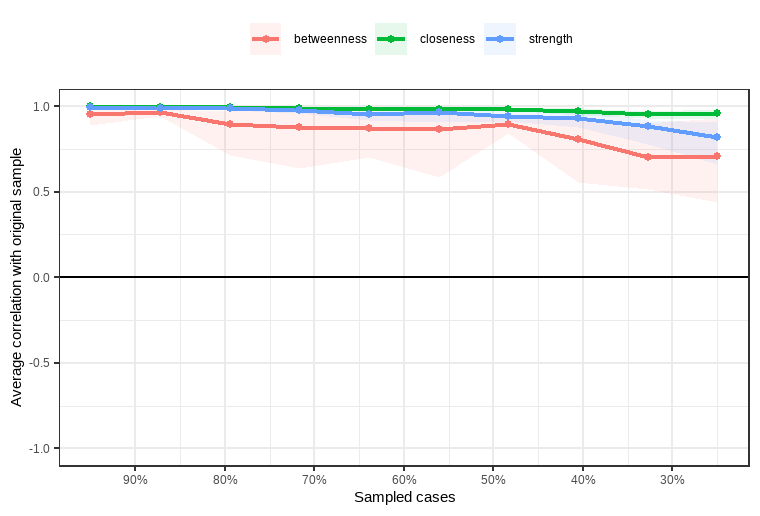


Supplementary Figure 1. Stability test of centrality indicators for each item (network subset bootstrap plot)

It shows that the centrality index of each item is relatively stable.


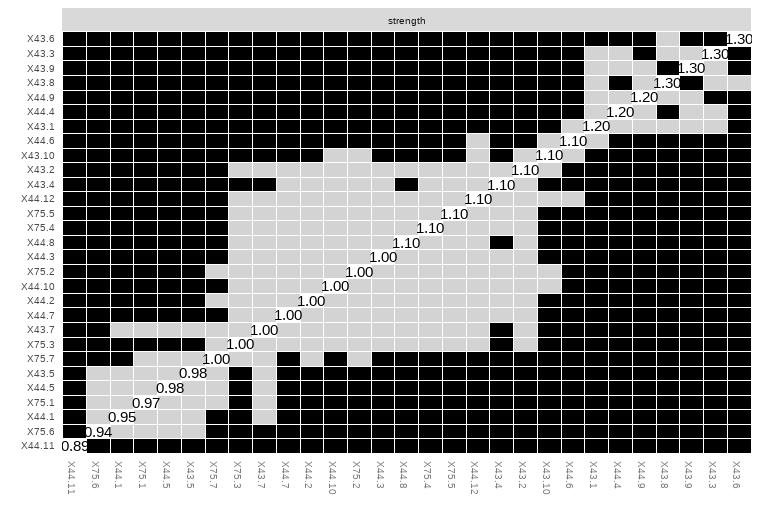


Supplementary Figure 2. Stability of node strength

There is a significant difference between the items, and the node strengths are stabilized for each of them.


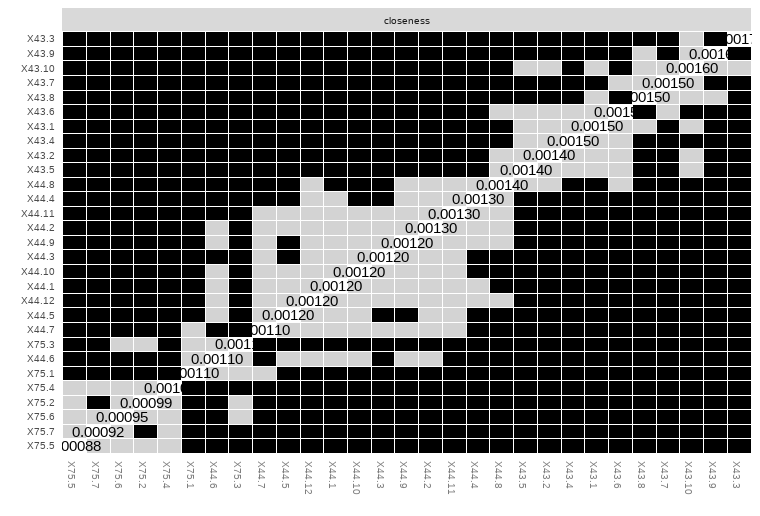


Supplementary Figure 3. Stability of node closeness

There are significant differences between the items, indicating that the closeness of the nodes is stabilized across topics.


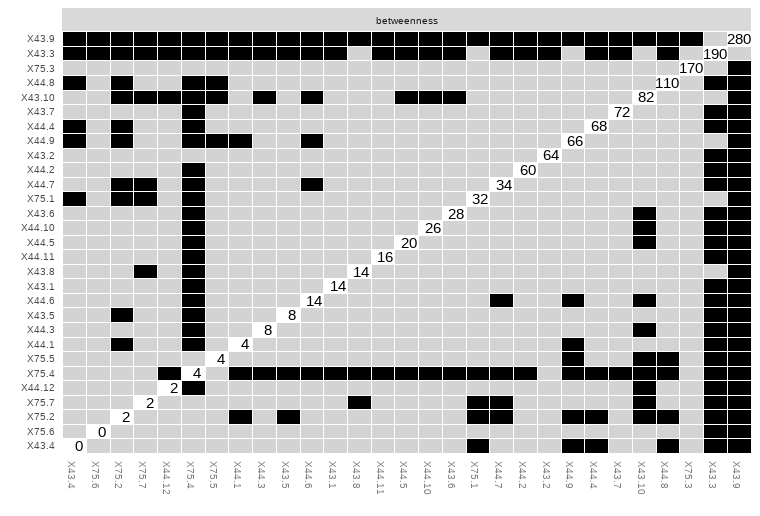


Supplementary Figure 4. Stability of Nodal Mediativity

There are differences between some of the topics, but some are significant and some are not, and the node betweenness varies across items depending on their differences.


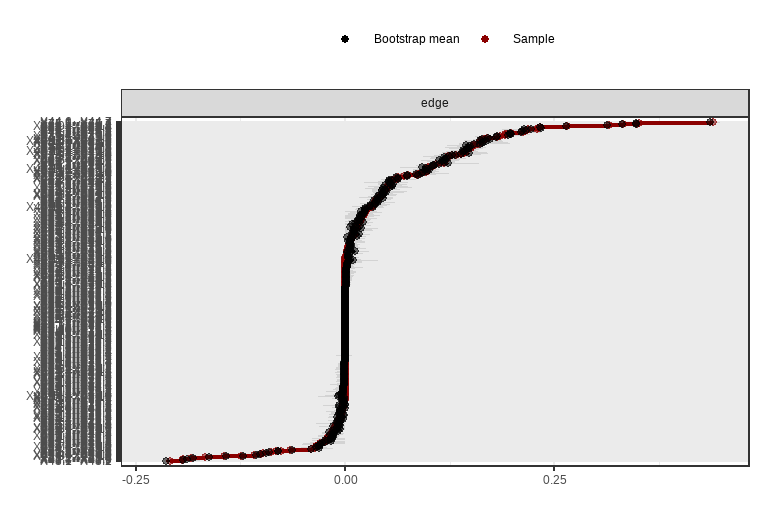


Supplementary Figure 5. Stability of edge weights

The stability of the edge weights was calculated by bootstrap method, and the fit was good, indicating the stability of the edge weights between the individual question items. Bootstrap confidence intervals for the edges were calculated and 95% confidence intervals were estimated using bootstrapping to test the variability of the edge weights. The black line in the figure represents the mean value derived from the Bootstrap method, and the red line reaches the mean value estimated from the original sample. The gray portion represents the confidence intervals corresponding to the two methods.


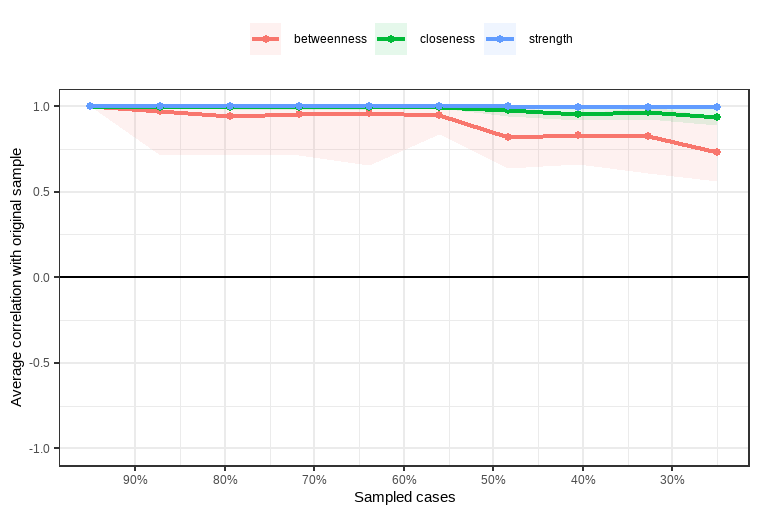


Supplementary Figure 6. Stability test of centrality indicators for each dimension (bootstrap diagram of network subsets)

It shows that the centrality indicators of each dimension are stable.


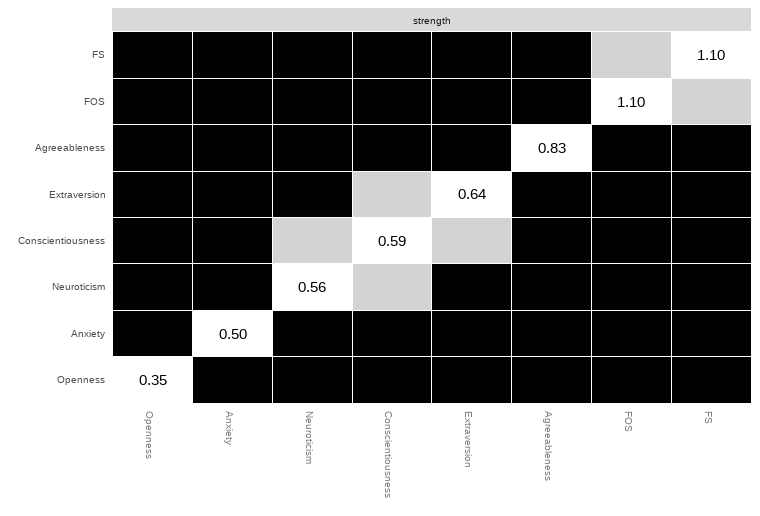


Supplementary Figure 7. Strength Stability of Dimensions

There is a significant difference between the dimensions, and the strength is stable between the dimensions.


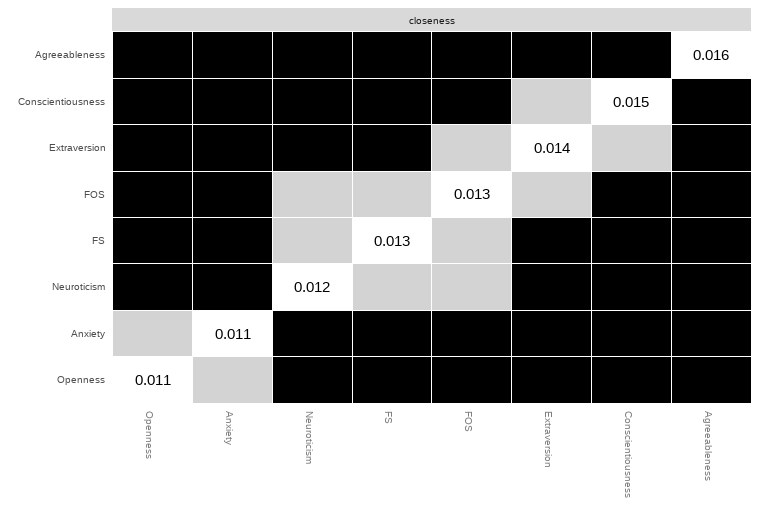


Supplementary Figure 8. Closeness Stability by Dimension

Significant differences between dimensions indicate closeness stability.


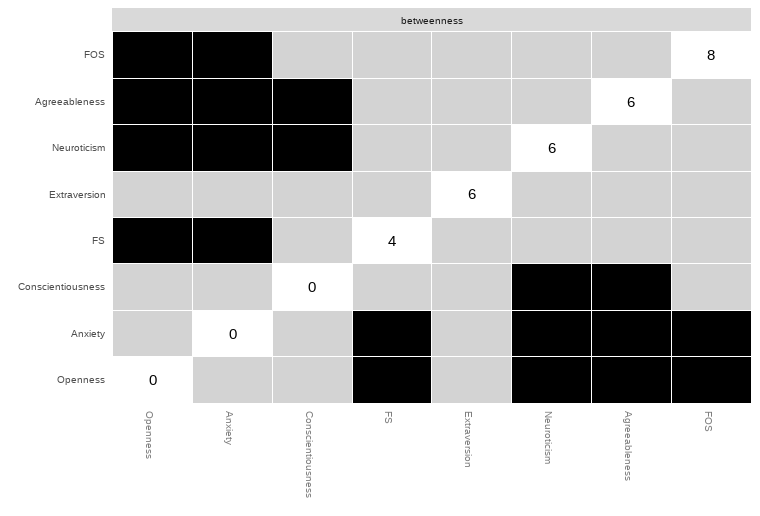


Supplementary Figure 9. Stability of betweenness across dimensions

There is a significant difference in the betweenness of the dimensions, and the betweenness stability is stable.


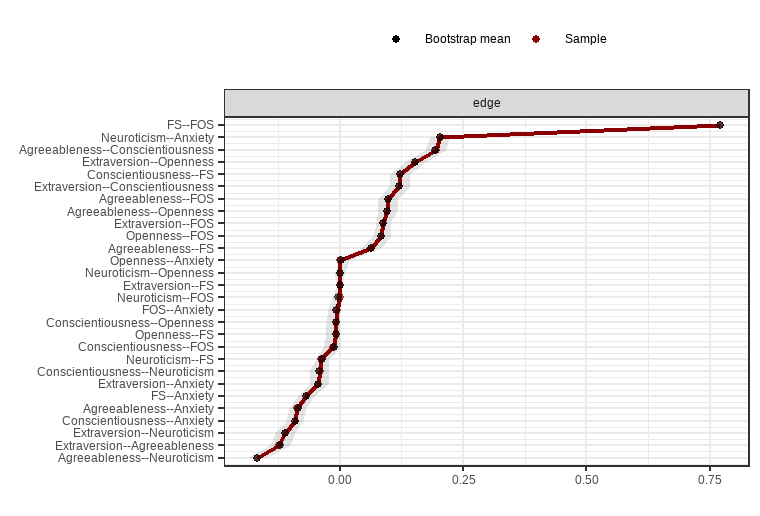


Supplementary Figure 10. Edge Weight Stability

The side weight accuracy is well-fitted and the edge weights are stable.


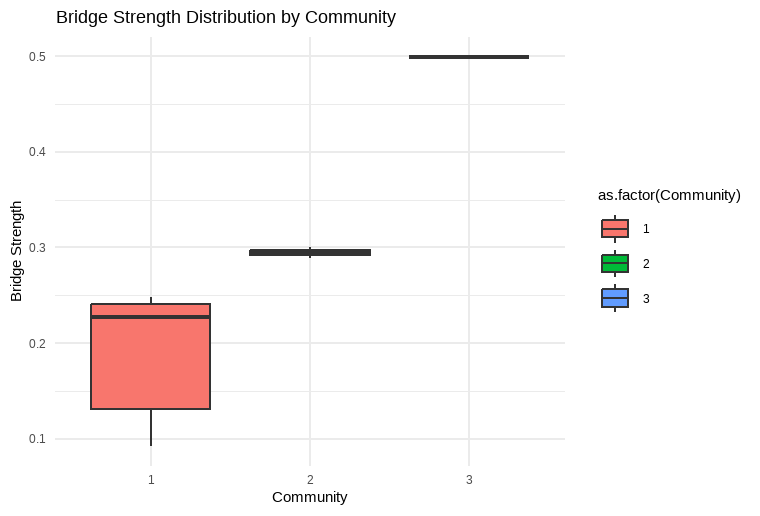


Supplementary Figure 11. Strength of community bridge connections

Visualization of the distribution of bridge connection strengths with the highest indicator of generalized anxiety bridge connections.
