# Supplementary material for: A network analysis study on the relationship between generalized anxiety symptoms, big five personality and perceived social support of Chinese residents during COVID-19
Source: Front Public Health. 2025 Feb 13;13:1548718. doi: 10.3389/fpubh.2025.1548718 (PMC11864928; doi:10.3389/fpubh.2025.1548718)
Supplement: Supplementary file 2 [file Supplementary_file_2.docx]

# Set E -------------------------------------------------------------------

install.packages("showtext")

install.packages("networktools")

library(showtext)

font_add("SimHei", "SimHei.ttf")

library(networktools)

showtext_auto()

install.packages(c("psych", "qgraph", "bootnet"))

install.packages("rgl", type = "source")

library(rgl)

library(psych)

library(qgraph)

library(bootnet)

library(ggplot2)

library(dplyr)

setwd("C:/Users/lenovo/Desktop/PBICR/数据分析") # 换自己的wd

#导入数据

PSSS <- read.csv("PSSS.csv")

BFI <- read.csv("BFI.csv")

GAD <- read.csv("GAD-7.csv")

#导出列名

a <- colnames(PSSS)

a <- a[2:13]

a[[1]] <- "X44. 在我遇到问题时.有些人.亲戚.邻居.同事.会出现在我身旁"

b <- colnames(BFI)

b <- b[2:11]

b[1] <- "X43. 我认为我话不多"

c <- colnames(GAD)

c <- c[2:8]

c[1] <- "X75. 感觉紧张.焦虑或急切"

#列名合集items

items <- c(a,b,c)

#合并数据

combined_df <- merge(PSSS, BFI, by = "序号")

combined_df <- merge(combined_df, GAD, by = "序号")

# 处理列名

n_X44 <- 12 # Adjust based on your actual number of X44 columns

n_X43 <- 10 # Adjust based on your actual number of X43 columns

n_X75 <- 7 # Adjust based on your actual number of X75 columns

# Rename columns for X44, X43, and X75

colnames(combined_df) <- c("序号", paste0("X44.", 1:n_X44),

paste0("X43.", 1:n_X43),

paste0("X75.", 1:n_X75))

# Change to factor --------------------------------------------------------

# Mapping for GAD-7

map_to_factor_GAD <- function(x) {

levels <- c("完全没有", "有几天", "超过一半", "几乎每天")

factor(x, levels = levels, labels = 1:length(levels))

}

# Mapping for BFI

map_to_factor_BFI <- function(x) {

levels <- c("非常不同意", "有点不同意", "既不同意也不反对", "有点同意", "非常同意")

factor(x, levels = levels, labels = 1:length(levels))

}

# Mapping for PSSS

map_to_factor_PSSS <- function(x) {

levels <- c("极不同意", "很不同意", "稍不同意", "中立", "稍同意", "很同意", "极同意")

factor(x, levels = levels, labels = 1:length(levels))

}

# Apply the mappings

combined_df <- combined_df %>%

mutate(across(starts_with("X75"), map_to_factor_GAD)) %>%

mutate(across(starts_with("X43"), map_to_factor_BFI)) %>%

mutate(across(starts_with("X44"), map_to_factor_PSSS))

# View the structure of the combined data frame

combined_df <- combined_df %>%

mutate(across(starts_with("X44"), as.numeric),

across(starts_with("X43"), as.numeric),

across(starts_with("X75"), as.numeric))

str(combined_df)

#反向记分

combined_df <- combined_df %>%

mutate(

X43.1 = 6 - X43.1,

X43.3 = 6 - X43.3,

X43.4 = 6 - X43.4,

X43.5 = 6 - X43.5,

X43.7 = 6 - X43.7

)

combined_df <- combined_df %>% select(c(2:30))

# 标准化数据

combined_df_scaled <- combined_df %>%

mutate(across(starts_with("X44"), scale),

across(starts_with("X43"), scale),

across(starts_with("X75"), scale))

# 查看标准化后的数据结构

str(combined_df_scaled)

#分类

traits = c("OS", "OS", "FS", "FS", "OS","OS", "OS", "FS", "OS", "OS","FS", "OS", "Extraversion", "Agreeableness", "Conscientiousness", "Neuroticism", "Openness", "Extraversion", "Agreeableness", "Conscientiousness", "Neuroticism", "Openness", "Anxiety", "Anxiety","Anxiety","Anxiety","Anxiety","Anxiety","Anxiety")

install.packages("corrplot")

library(corrplot)

M<-cor(combined_df)

View(M)

correlation_df <- as.data.frame(M)

judge_correlation <- function(r) {

if (abs(r) >= 0.6) {

return("强相关")

} else if (abs(r) >= 0.4 & abs(r) < 0.6) {

return("中相关")

} else {

return("弱相关")

}

}

M<-cor(combined_df)

correlation_matrix<-M

# 创建一个矩阵来存储相关性强度的判断结果

correlation_strengths_matrix <- matrix(NA, nrow(correlation_matrix), ncol(correlation_matrix))

diag(correlation_strengths_matrix) <- NA # 对角线元素设置为NA（因为自身与自身的相关性总是1）

# 遍历非对角线元素，判断相关性强度

for (i in 1:(nrow(correlation_matrix) - 1)) {

for (j in (i + 1):ncol(correlation_matrix)) {

r <- correlation_matrix[i, j]

correlation_strengths_matrix[i, j] <- judge_correlation(r)

correlation_strengths_matrix[j, i] <- correlation_strengths_matrix[i, j] # 因为相关矩阵是对称的

}

}

# 将相关性强度信息转换为向量（可选，用于统计）

correlation_strengths <- as.vector(correlation_strengths_matrix)

correlation_strengths <- correlation_strengths[!is.na(correlation_strengths)]

# 统计每种强度出现的次数和比例

strength_table <- table(correlation_strengths)

strength_proportion <- prop.table(strength_table)

# 打印结果

print("相关性强度统计：")

print(strength_table)

print("相关性强度比例：")

print(strength_proportion)

corrplot.mixed(M,tl.pos=c("lt"),

number.cex=0.2)

# Network analysis --------------------------------------------------------

network <- estimateNetwork(combined_df, default = "EBICglasso", corMethod = "spearman")

custom_colors <- c("#E69F00", "#56B4E9", "#009E73", "#F0E442", "#0072B2", "#D55E00","#CC79A7","#C6B9D3")

View(network)

getWmat(network)

adj.ggm<-getWmat(network)

sum(adj.ggm[upper.tri(adj.ggm,diag=F)]!=0)

total_weight <- sum(adj.ggm[adj.ggm != 0])

num_edges <- sum(adj.ggm[upper.tri(adj.ggm, diag = FALSE)] != 0)

average_weight <- total_weight / num_edges

num_edges <- network::nedges(network)

print(paste("Number of edges (using attribute):", num_edges))

View(num_edges)

#圆圈图

plot(network,

layout = "circle",

groups = traits,

negDashed = T,

color = custom_colors

)

#经典图

plot(network,

layout = "spring",

groups = traits,

negDashed = T,

color = custom_colors,

#legend.mode = "style2",

legend.cex = 0.5,

#nodeNames = items,

font = 2

)

#中心指标

centralityPlot(network, include = "all",scale="z-scores")

# 中心性指标稳定性

central_stability <- bootnet(network, nBoots = 100, type = "case", statistics = c("edge","strength", "closeness", "betweenness"))

corStability(central_stability)

#Drop 图

plot(central_stability, statistics = c("strength", "closeness", "betweenness"))

# 边权重准确性，不要type = case

central_stability1 <- bootnet(network, nBoots = 10, statistics = c("edge","strength", "closeness", "betweenness"))

central_stability1

plot(central_stability1, "strength", order = "sample")

plot(central_stability1, "closeness", order = "sample")

plot(central_stability1, "betweenness", order = "sample")

plot(central_stability1, "edge", order = "sample")

# 分组Network analysis ------------------------------------------------------

# 创建新列并根据分组规则求和

combined_df$Extraversion <- rowSums(combined_df[, c("X43.1", "X43.6")])

combined_df$Agreeableness <- rowSums(combined_df[, c("X43.2", "X43.7")])

combined_df$Conscientiousness <- rowSums(combined_df[, c("X43.3", "X43.8")])

combined_df$Neuroticism <- rowSums(combined_df[, c("X43.4", "X43.9")])

combined_df$Openness <- rowSums(combined_df[, c("X43.5", "X43.10")])

combined_df$FS <- rowSums(combined_df[, c("X44.3", "X44.4", "X44.8", "X44.11")])

combined_df$FOS <- rowSums(combined_df[, c("X44.1", "X44.2", "X44.5", "X44.6", "X44.7", "X44.9", "X44.10", "X44.12")])

combined_df$Anxiety <- rowSums(combined_df[, paste0("X75.", 1:7)])

# 查看新的数据框架

head(combined_df)

combined_df <- combined_df %>% select(c(30:37))

install.packages("corrplot")

library(corrplot)

M<-cor(combined_df)

View(M)

correlation_df <- as.data.frame(M)

judge_correlation <- function(r) {

if (abs(r) >= 0.6) {

return("强相关")

} else if (abs(r) >= 0.4 & abs(r) < 0.6) {

return("中相关")

} else {

return("弱相关")

}

}

M<-cor(combined_df)

correlation_matrix<-M

# 创建一个矩阵来存储相关性强度的判断结果

correlation_strengths_matrix <- matrix(NA, nrow(correlation_matrix), ncol(correlation_matrix))

diag(correlation_strengths_matrix) <- NA # 对角线元素设置为NA（因为自身与自身的相关性总是1）

# 遍历非对角线元素，判断相关性强度

for (i in 1:(nrow(correlation_matrix) - 1)) {

for (j in (i + 1):ncol(correlation_matrix)) {

r <- correlation_matrix[i, j]

correlation_strengths_matrix[i, j] <- judge_correlation(r)

correlation_strengths_matrix[j, i] <- correlation_strengths_matrix[i, j] # 因为相关矩阵是对称的

}

}

# 将相关性强度信息转换为向量（可选，用于统计）

correlation_strengths <- as.vector(correlation_strengths_matrix)

correlation_strengths <- correlation_strengths[!is.na(correlation_strengths)]

# 统计每种强度出现的次数和比例

strength_table <- table(correlation_strengths)

strength_proportion <- prop.table(strength_table)

# 打印结果

print("相关性强度统计：")

print(strength_table)

print("相关性强度比例：")

print(strength_proportion)

corrplot.mixed(M,tl.pos=c("lt"),

number.cex=0.4)

network <- estimateNetwork(combined_df, default = "EBICglasso", corMethod = "spearman")

custom_colors <- c("#CC79A7", "#E69F00", "#56B4E9", "#009E73", "#F0E442", "#0072B2", "#D55E00", "#8E44AD")

traits = colnames(combined_df)

View(network)

str(combined_df)

getWmat(network)

adj.ggm<-getWmat(network)

sum(adj.ggm[upper.tri(adj.ggm,diag=F)]!=0)

total_weight <- sum(adj.ggm[adj.ggm != 0])

num_edges <- sum(adj.ggm[upper.tri(adj.ggm, diag = FALSE)] != 0)

average_weight <- total_weight / num_edges

#圆圈图

plot(network,

layout = "circle", # 圆形布局

groups = traits, # 节点的分组方式

label.cex = 1.0, # 节点标签的大小

label.color = 'black', # 节点标签的颜色

negDashed = TRUE, # 负边使用虚线表示

legend = TRUE, # 显示图例

color = custom_colors, # 自定义颜色

legend.cex = 0.5, # 图例的大小

legend.mode = 'style3', # 图例的样式

nodeNames = colnames(data),# 节点名称

font = 2, # 标签字体样式

palette = "ggplot2") # 使用ggplot2调色板

plot(network,

layout = "circle",

groups = traits,

negDashed = T,

color = custom_colors,

legend.cex = 0.5,

#nodeNames = items,

font = 2

)

#经典图

plot(network,

layout = "spring",

groups = traits,

negDashed = T,

color = custom_colors,

legend.cex = 0.5,

#nodeNames = items,

font = 2

)

# 画中心性图

centralityPlot(network, orderBy = "Betweenness", include = "all", scale = "z-scores")

centralityPlot(network, orderBy = "Betweenness", include = "all")

# bridge

bridge_sym <- bridge(network$graph, communities = c("1","1","1","1","1","2","2","3"))

bridge_sym

#画bridge strength

plot(bridge_sym, order = "value", include = c('Bridge Strength'))

# 进行Bootstrap抽样以评估中心性指标的稳定性

# 中心性指标稳定性

central_stability <- bootnet(network, nBoots = 100, type = "case", statistics = c("edge","strength", "closeness", "betweenness"))

corStability(central_stability)

# 计算中心稳定性系数

cs_results <- corStability(central_stability)

# 打印中心稳定性系数结果

print(cs_results)

# 绘制中心性指标的稳定性图

plot(central_stability, statistics = c("strength", "closeness", "betweenness"))

# 边权重准确性，不要type = case

central_stability1 <- bootnet(network, nBoots = 500, statistics = c("edge","strength", "closeness", "betweenness", "bridgeStrength"),communities = c("1","1","1","1","1","2","2","3"))

central_stability1

plot(central_stability1, "strength", order = "sample")

plot(central_stability1, "closeness", order = "sample")

plot(central_stability1, "betweenness", order = "sample")

plot(central_stability1, "edge", order = "sample")

# 提取桥接强度结果

#创建社区

communities = c("1","1","1","1","1","2","2","3")

#计算桥接中心性

bridge_sym <- bridge(network$graph, communities = c("1","1","1","1","1","2","2","3"))

plot(bridge_sym, order = "value", include = c('Bridge Strength'))

#节点Bridge strength

bridge_strength <- bridge_sym$`Bridge Strength`

bridge_strength #节点最大的是Anxiety，其实和上面一样

# 创建一个数据框来存储结果

bridge_df <- data.frame(

Variable = colnames(combined_df),

Community = communities,

BridgeStrength = bridge_strength

)

# 按社区分组并计算平均桥接强度

avg_bridge_strength <- aggregate(BridgeStrength ~ Community, data = bridge_df, FUN = mean)

# 打印平均桥接强度

print(avg_bridge_strength)

# 可视化桥接强度的分布

ggplot(bridge_df, aes(x = as.factor(Community), y = BridgeStrength, fill = as.factor(Community))) +

geom_boxplot() +

labs(x = "Community", y = "Bridge Strength", title = "Bridge Strength Distribution by Community") +

theme_minimal()

#性别网络比较

install.packages("bruceR")

install.packages("NetworkComparisonTest")

install.packages(c("psych", "qgraph", "bootnet"))

install.packages("Networktools")

install.packages("showtext")

library(showtext)

library(qgraph)

library(ggplot2)

library("bruceR")

library("NetworkComparisonTest")

library("networktools")

setwd("C:/Users/lenovo/Desktop/August - 副本")

Male<-import("Male.xlsx")

Female<-import("Female.xlsx")

map_to_factor_Male <- function(x) {

levels <- c("完全没有", "有几天", "超过一半", "几乎每天")

factor(x, levels = levels, labels = 1:length(levels))

}

# Mapping for Male

map_to_factor_Male <- function(x) {

levels <- c("非常不同意", "有点不同意", "既不同意也不反对", "有点同意", "非常同意")

factor(x, levels = levels, labels = 1:length(levels))

}

# Mapping for Male

map_to_factor_Male <- function(x) {

levels <- c("极不同意", "很不同意", "稍不同意", "中立", "稍同意", "很同意", "极同意")

factor(x, levels = levels, labels = 1:length(levels))

}

# Apply the mappings

Male <- mutate(Male, across(starts_with("X75"), map_to_factor_GAD))

Male <- mutate(Male, across(starts_with("X43"), map_to_factor_BFI))

Male <- mutate(Male, across(starts_with("X44"), map_to_factor_PSSS))

Male <- Male %>%

mutate(across(starts_with("X75"), map_to_factor_GAD)) %>%

mutate(across(starts_with("X43"), map_to_factor_BFI)) %>%

mutate(across(starts_with("X44"), map_to_factor_PSSS))

# View the structure of the combined data frame

Male <- Male %>%

mutate(across(starts_with("X44"), as.numeric),

across(starts_with("X43"), as.numeric),

across(starts_with("X75"), as.numeric))

str(Male)

Male <- Male %>%

mutate(

X43.1 = 6 - X43.1,

X43.3 = 6 - X43.3,

X43.4 = 6 - X43.4,

X43.5 = 6 - X43.5,

X43.7 = 6 - X43.7

)

Male_scaled <- Male %>%

mutate(across(starts_with("X44"), scale),

across(starts_with("X43"), scale),

across(starts_with("X75"), scale))

map_to_factor_Female <- function(x) {

levels <- c("完全没有", "有几天", "超过一半", "几乎每天")

factor(x, levels = levels, labels = 1:length(levels))

}

# Mapping for Female

map_to_factor_Female <- function(x) {

levels <- c("非常不同意", "有点不同意", "既不同意也不反对", "有点同意", "非常同意")

factor(x, levels = levels, labels = 1:length(levels))

}

# Mapping for Female

map_to_factor_Female <- function(x) {

levels <- c("极不同意", "很不同意", "稍不同意", "中立", "稍同意", "很同意", "极同意")

factor(x, levels = levels, labels = 1:length(levels))

}

# Apply the mappings

Female <- Female %>%

mutate(across(starts_with("X75"), map_to_factor_GAD)) %>%

mutate(across(starts_with("X43"), map_to_factor_BFI)) %>%

mutate(across(starts_with("X44"), map_to_factor_PSSS))

# View the structure of the combined data frame

Female <- Female %>%

mutate(across(starts_with("X44"), as.numeric),

across(starts_with("X43"), as.numeric),

across(starts_with("X75"), as.numeric))

str(Female)

Female <- Female %>%

mutate(

X43.1 = 6 - X43.1,

X43.3 = 6 - X43.3,

X43.4 = 6 - X43.4,

X43.5 = 6 - X43.5,

X43.7 = 6 - X43.7

)

Female_scaled <- Female %>%

mutate(across(starts_with("X44"), scale),

across(starts_with("X43"), scale),

across(starts_with("X75"), scale))

gender_compare<-NCT(Male,Female,gamma=0.5,it=1000,

binary.data=FALSE,

test.edges = TRUE, edges="all",

test.centrality = TRUE,centrality = c("strength"),

p.adjust.methods = "BH",

nodes="all")

save(gender_compare,file="gender_compare.Rdata")

summary(gender_compare)

#global strength results全局强度结果

gender_compare$glstrinv.pval

gender_compare$glstrinv.real

plot(gender_compare,what="strength",labels = sprintf("%.2f", df$pvalue))

#network test results网络结构比较结果

gender_compare$nwinv.pval

gender_compare$nwinv.real

plot(gender_compare,what="network",labels = sprintf("%.2f", df$pvalue))

#edge results边线比较结果

gender_compare$einv.pval

sum(gender_compare$einv)
